# Oral health status and perceived barriers to care among older adults in Alabama, United States: an observational community case study of prevalence and associated risk factors

**DOI:** 10.3389/fpubh.2026.1752129

**Published:** 2026-03-13

**Authors:** Nathan R. Smith, Anastasia M. Hartzes, Raquel Mazer, Joana Cunha-Cruz

**Affiliations:** 1 Department of Clinical and Community Sciences, School of Dentistry, University of Alabama at Birmingham, Birmingham, AL, United States; 2 Department of Biostatistics, School of Public Health, University of Alabama at Birmingham, Birmingham, AL, United States

**Keywords:** evidence-based public health, access to care, barriers to care, dental, disparity, geriatrics (GER), surveillance

## Abstract

**Objective:**

Limited data exists on the oral health status of the older adult population of Alabama. Improved surveillance programs are needed for estimations and planning.

**Aim:**

This study describes oral health status and barriers to routine dental services utilization among older adults in central Alabama.

**Methods:**

A retrospective analysis was conducted using observational surveillance data from older adults attending senior centers and senior living communities. Data included demographics, oral care behaviors, care utilization and clinical findings from intraoral screenings. Data were summarized using descriptive statistics, and regression models were used to investigate which factors were associated with oral health status (edentulism and untreated dental caries), routine dental care, and oral care needs.

**Results:**

566 respondents were assessed: mean age 73.2, 70.0% female, and most identified as Black or African American (58.6%) or White (39.6%). The prevalence of edentulism was 28.3%, and 56.6% of dentate participants had untreated decay. Edentulous participants were more likely to have longer intervals since their last dental visit. Dentate participants were more likely to require early or urgent care. Untreated decay was significantly associated with Black/African American race, inability to drive, and lack of dental insurance. Younger age and receiving routine care in the past three years were associated with greater retention of natural teeth. Most participants did not visit a dentist in the previous year, citing cost, lack of perceived need, or other priorities. There were no differences in dentate status regarding emergency dental visits or regular dental provider status.

**Conclusions:**

These findings underscore the need for expanded access to oral health services and targeted interventions for older adults in Alabama, especially those facing socioeconomic and mobility barriers. The academic-public health partnership successfully identified key oral health disparities and barriers, providing actionable insights to inform future programmatic and policy efforts for older adults in Alabama.

## Introduction

Oral health is essential to overall health and wellbeing at all ages. Social determinants of health and barriers to care—including lack of transportation, financial burden, underinsurance or the lack of insurance, limited oral health literacy, and fear—contribute to the lack of routine dental services which can result in the need for more advanced, costly treatments (1). Population projections for the United States show the population over the age of 65 continuing to increase and surpassing the population under the age of 18 by the 2030s (2–5). Multiple chronic conditions, polypharmacy, and decreased mobility impact oral care (6–8). As age increases, oral health care utilization declines while prevalence of oral disease (i.e., caries, periodontal disease, cancer) increases (6, 9–11). Frail or institutionalized older adults are disproportionately affected by oral disease (12). Physical and mental disability contribute to active oral disease and the eventual need for acute care (13, 14). Declining oral health practices and dental visits over a lifespan, especially for those with complex medical conditions, has been observed (15, 16).

Limited data exists on the oral health status of the older adult population of Alabama; (17) surveillance programs are needed for estimations and planning. Alabama counties are socially vulnerable and health disparities are widespread (18). Approximately 25% of Alabamians live in rural areas and 15% are considered non-institutionalized disabled (19). In 2021, Alabama ranked the 3^rd^ lowest in per capita income, 25% less than the national average, and 18.3% of its population is 65 and older when compared to 17.7% in the US. Alabama's overall poverty rate is 15% with substantially higher poverty among Black/African American (29%) and Latino (32%) residents (20, 21). In 2020, 61% of Alabama adults (18 years and older) had a dental visit the prior year, lower than the national rate of 66%, and 49% had at least one tooth extracted, exceeding the national rate (42%) (22). All but two counties in Alabama are dental health professional shortage areas (HPSA) (23). Therefore, in Alabama, there is a need to better understand the oral health needs of older adults.

The purpose of this work is to conduct an investigational and exploratory analysis of public health surveillance data to describe the oral health statuses and identify barriers to routine preventive dental care among older adults living in central Alabama. These findings will help determine the potential need for future interventions, future investigation, and resources targeted to improving oral health among the older adult population.

### Context (setting and population)

Participants: Adults (60-years-old and older) attending senior centers or living in senior living communities in Birmingham city area and neighboring counties in central Alabama. Sites were located within a 60-minute driving time from Birmingham, Alabama.

### Key programmatic detail

The UAB School of Dentistry has partnered with the Alabama Department of Senior Services (ADSS) to conduct oral health screenings of adults age 60 years and older (hereinafter, older adults) for over 8 years. This program is designed to promote oral health awareness, enhance oral health literacy and fluency, determine the oral health status, and assess needs and barriers to care among older adults. UAB engages dental students and faculty to educate, assess, and provide resources and referrals for dental services. To reach a greater number of participants at a time when many senior centers remained closed due to COVID-19 pandemic precautions, these activities were expanded to a limited number of senior living communities in Jefferson County in 2021 as a means to promote oral health and engage with older adults while maintaining safety.

**Data collection:** Data was collected through a questionnaire delivered in-person and an oral health screening administered by clinical faculty and a team of third- and fourth-year dental students. The oral health screening tool utilized is based on the Basic Screening Survey for Older Adults published by the Association of State and Territorial Dental Directors (ASTDD) and revised in 2018 (24) in which oral screening variables and examiner calibration are described. The oral health screening is an oral health assessment that results in a recommendation for routine, early, or urgent care. In summary, the oral screenings were conducted by visual inspection, no magnification or dental explorer hand instruments were utilized; a visually obvious carious break in enamel was recorded as untreated decay; only natural teeth (not implants or pontics of fixed bridges) were counted as existing teeth. Treatment urgency was assigned based on the following criteria: (1) no obvious problems was equated to routine care; (2) pain, infection, or suspicious soft tissue lesion were equated to urgent care; and (3) all other less urgent needs were equated to early care.

Study data were recorded and managed using REDCap (Research Electronic Data Capture) electronic data capture tools hosted at UAB Department of Medicine IT (DOM IT (25, 26). REDCap is a secure, web-based software platform designed to support data capture for research studies, providing (1) an intuitive interface for validated data capture; (2) audit trails for tracking data manipulation and export procedures; (3) automated export procedures for seamless data downloads to common statistical packages; and (4) procedures for data integration and interoperability with external sources. Following the data collection conducted between 2017 and 2022, the data was de-identified and used for public health surveillance activities; data were not stratified by collection year and were aggregated for analytic purposes. No data were collected during the 2020 calendar year.

### Outcome variables

Participants were asked about engagement with dental services, including frequency of dental visits per year, time since the last visit at the time of screening (2017–2022), and type of services received. Of interest were the following outcome measures:

1. Routine care received in the previous 3 years (yes/no), defined as routine checkup and cleaning.
2. Untreated decay (yes/no).
3. Total number of natural teeth remaining (0, 1–19, 20–32 teeth), grouped post-collection.
4. Treatment urgency (urgent or early care, no obvious problem).

Care and service-related questions were self-reported by the participant; all other outcomes were based on clinical oral screening.

### Covariates

Demographic variables included age (years), race (African American or Black, White, Other), marital status (married, single/never married, separated/divorced/other, widowed), highest level of education received (grades 1–11, high school or GED, some college or associate's degree, bachelor's degree or higher), monthly income (< $1500/month, $1500 or more/month), income source (Social Security (SS), pension (no SS), employment + other source), having any form of dental insurance (yes/no), screening location (living facility, senior center), county of residence, and if the participant still drives (yes/no). The county of residence was classified according to Rural-Urban Continuum Codes (RUCC) for 2013, which uses the population of the metropolitan (metro) area and closeness to an urban area to define counties (27). The 2013 classification was applied as it most closely matches the data collection period. The RUCC are then grouped according to being a metro-area (*RUCC* = 1, 2, 3), non-metro but urban adjacent (*RUCC* = 4, 6, 8; generally representing suburban areas), and non-metro (*RUCC* = 5, 7, 9; representing rural areas). For analytic purposes, we dichotomized the RUCC by retaining the metro classification (*RUCC* = 1, 2, 3) and combining the two non-metro areas (*RUCC* = 4, 5, 6, 7, 8, 9) to a single non-metro group for interpretability.

Clinical oral screening variables included number of maxillary, mandibular and total natural teeth, edentulous (yes/no), untreated decay (yes/no), root fragment (yes/no), need for periodontal care (yes/no), removable upper and lower dentures (yes/no), and treatment urgency (urgent care, early care, no obvious problem); variable descriptions and examiner calibration is described by the ASTDD materials (24). Self-reported variables included time since last dental visit (never, past 12 months, 1–2 years, 2–5 years, 5 or more years), if they visited the dentist in the last 3 years for routine care (yes, no, unsure), and if they visited the dentist in the 12 months for routine care (yes/no).

Missing data varied by participant and variable, and are noted in summary tables. One participant's responses were excluded from the clinical variables (periodontal care, root fragments, untreated decay and number of teeth) due to inconsistent edentulous identification that could not be adjudicated.

### Statistical analysis

Data were summarized using descriptive statistics. Geography and edentulous status groups were compared using Chi-square test for proportions, t-test or Wilcoxon, as appropriate for means or medians.

Logistic regression was performed to identify factors (including potential barriers) associated with the outcome variables as specified above: (1) receiving dental cleaning/routine care in the previous 3 years (binary logistic); (2) untreated decay (binary logistic); (3) number of natural teeth remaining (ordinal logistic); and (4) treatment urgency (binary logistic). Therefore, treatment urgency was analyzed as a dichotomous outcome, such that Early Care and Urgent Care were combined to a single group, compared to No Obvious Problem due to low frequency of participants identified as requiring Urgent Care (*N* = 39 out of the whole sample). Variables for the full models included age, race, sex, marital status, screening site, driving status, region of residence, health insurance status, monthly income, dental insurance, dentate status (when appropriate); and highest education received, including all interactions. Due to low frequency of the reported “other” category, these participants were excluded for analytic purposes; therefore, race was analyzed as dichotomous (Black or African American (AA), White). Variables included in the final models were selected using stepwise selection and the model performance was evaluated using Hosmer and Lemeshow goodness of fit test, Akaike Information Criterion (AIC), assessment of overall model significance, interpretability, and significance of individual predictors. The ordinal logistic model was evaluated for meeting proportional odds assumption, modeling the following groups of natural teeth remaining: 0 teeth (complete tooth loss), 1–19 teeth (moderate to severe tooth loss), and 20–32 teeth (functional dentition (28, 29)).

All analyses were performed using SAS v9.4, Cary, N.C, with statistical significance defined as α = 0.05.

## Results

### Sample description

Data were available for approximately 566 older adults attending senior centers or living in senior living communities in the Birmingham, Alabama area, representing 23 neighboring counties (Table 1). The average age of respondents was 73.2 years (SD=8.1); most were female (70.0%), and more participants were Black or African American (58.6%) compared to other groups. Fewer participants were married (16.9%) compared to single/never married (24.5%), separated/divorced (28.3%), or widowed (30.3%).

**Table 1:** Description of sample (*N* = 566).

			**Dentate status**		
**Characteristic**, ***n (%) unless noted***	**Level**	**All**	**Dentate 400 (71.7)**	**Edentulous 158 (28.3)**	***p*** **-value** ^†^
Gender	Female	396 (70.0)	271 (67.8)	119 (75.3)	0.0757
	Male	170 (30.0)	129 (32.3)	39 (24.7)	
Marital status (*n* = 551)	Married	93 (16.9)	75 (19.4)	16 (10.2)	**0.0292**
	Single, never married	135 (24.5)	93 (24.1)	40 (25.5)	
	Separated / Divorced/ Other	156 (28.3)	111 (28.8)	44 (28.0)	
	Widowed	167 (30.3)	107 (27.7)	57 (36.3)	
Race (*n* = 555)	Black/African American	325 (58.6)	215 (55.1)	105 (66.9)	**0.0219**
	White	220 (39.6)	166 (42.6)	51 (32.5)	
	Other	10 (1.8)	9 (2.3)	1 (0.6)	
Screening location	Living facility	242 (42.8)	164 (41.0)	74 (46.8)	0.2102
	Senior center	324 (57.2)	236 (59.0)	84 (53.2)	
Income (*n* = 491)	< $1500/month	194 (39.5)	198 (57.4)	94 (68.1)	**0.0281**
	$1500 or more/month	297 (60.5)	147 (42.6)	44 (31.9)	
Income Source (*n* = 539)^a^	SS/SSD^b^ (may have other source)	493 (91.5)	336 (89.4)	149 (96.8)	**0.0102**
	Pension/Other (no SS)	21 (3.9)	19 (5.1)	2 (1.3)	
	Employment + other source	24 (4.5)	21 (5.6)	3 (2.0)	
Dental Insurance (*n* = 563)	No	331 (61.8)	224 (59.7)	101 (65.6)	0.2071
	Yes	205 (38.3)	151 (40.3)	53 (34.4)	
Health w/Dental (*n* = 484)^c^	No	262 (54.1)	191 (56.2)	67 (49.3)	0.1720
	Yes	222 (45.9)	149 (43.8)	69 (50.7)	
Still driving (*n* = 546)	No	189 (34.6)	117 (30.6)	68 (43.6)	0.0044
	Yes	357 (65.4)	265 (69.4)	88 (56.4)	
Any disability (*N* = 540)	No	252 (46.7)	182 (48.0)	66 (43.1)	0.3061
	Yes	288 (53.3)	197 (52.0)	87 (56.9)	
Disability type (*N* = 254)^d^	Visual	40 (15.8)	28 (15.6)	11 (15.3)	0.9525
	Ambulatory	33 (13.0)	24 (13.4)	9 (12.5)	
	Hearing	34 (13.4)	26 (14.5)	8 (11.1)	
	Cognitive	13 (5.1)	9 (5.0)	4 (5.6)	
	2 or more limitations	134 (52.8)	92 (51.4)	40 (55.6)	
Highest education attained (*n* = 546)	Grades 1–11	136 (24.9)	92 (23.8)	43 (28.5)	**0.0205**
	High school diploma or GED	213 (39.0)	142 (36.7)	67 (44.4)	
	Some college or associate's degree	139 (25.5)	105 (27.1)	33 (21.9)	
	Bachelor's degree or higher	58 (10.6)	48 (12.4)	8 (5.3)	
Year of data collection (*N* = 566)	2017–2019	176 (31.1)	140 (35.0)	34 (21.5)	**0.0015**
	2021–2022	390 (68.9)	260 (65.0)	124 (78.5)	
RUCC Region of residence^e^	Metro	365 (64.5)	259 (64.8)	100 (63.3)	0.7462
	Non-metro	201 (35.5)	141 (35.3)	58 (36.7)	
Age (years)	*Mean (SD)*	73.2 (8.1)	72.6 (7.9)	74.7 (8.4)	**0.0072** ^**f**^
	*Median (min, max)*	72 (60, 97.0)	71.0 (60.0, 97.0)	74.0 (60.0, 96.0)	
RUCC (*n* = 566)	*Median (min, max)*	3 (1, 9)	3.0 (1, 9)	3.0 (1, 9)	0.3852^g^

^a^Participant responded with “no income”.

^b^Social Security.

^c^Health insurance provides dental benefits; 7 respondents answered “Unknown”.

^d^Of those who answered “yes” to having a disability.

^e^According to the Urban-Rural Continuum.

^†^Likelihood ratio Chi-square test, unless otherwise noted; bold p-values indicate statistical significance at α= 0.05.

^f^Pooled t-test.

^g^Wilcoxon test, t-approximation.

More participants were screened in a senior center (57.2%) overall; those in a metro area tended to be screened in a living facility (61.4%), whereas those in an urban-adjacent area tended to be screened in a senior center (95.6%). All non-metro participants were screened in a senior center. Most participants had a monthly income of $1500 or more (60.5%). Most participant's main income source was tied to Social Security (SS, 91.5%). Over one-third of participants (38.3%) reported having some form of dental insurance, and more did not report having health insurance with dental benefits (54.1%) than did. Most participants reported still driving personal vehicles (65.4%); and most participants had at least a high school education or GED (75.1%).

Related to their natural dentition, most participants were dentate (71.7%), compared to those who were edentulous (28.3%). Dentate status did not differ by gender (*p* > 0.05). However, in this data sample, those who were Black or African American were more likely to be edentulous compared to White participants. Edentulism was observed in widowed individuals at 36.3% versus 27.7% who were dentate, and fewer married participants were edentulous (10.2% versus 19.4%); all other groups were similar by dentate status. Decreased monthly income was consistent with increased chance of being edentulous; specifically, of those reporting a monthly income of less than $1500 per month, 68.1% were edentulous. By majority, participants reported relying on Social Security as the predominant source of income (91.5%) and of those who were edentulous, relied primarily on Social Security as a form of income (96.8%). Those who did not drive were more likely to be edentulous (43.6%) compared to those who did. Those with some college or higher education attainment were more likely to be dentate; while those with a high school diploma/GED were more likely to be edentulous; those whose education stopped in Grades 1–11 did not substantially differ. On average, edentulous participants were older (Mean = 74.7, SD = 8.4) than dentate (Mean = 72.6, SD = 7.9). More participants in the 2021–2022 collection period were edentulous compared to the 2017–2019 period.

Dentate status did not differ by screening site, having dental insurance at all or through health insurance, having a functional limitation at all or by type, or by RUCC region of residence (all *p* > 0.05, Table 1).

**Research question 1** *: What are the oral health status and dental service utilization among older adults in Birmingham and central Alabama?*

In the older adult Alabamian sample assessed, 64.5% resided in metropolitan regions and 53.3% reported a disability, with no significant differences observed between dentate and edentulous groups for these variables. Significant differences were found for other factors. More participants had at least one tooth (*N* = 400 (71.7%), Dentate) than were edentulous (*N* = 158 (28.3%), Edentulous).

When evaluating their use of prostheses for replacement of lost dentition, removable upper dentures were used by 48.5% of participants overall, with greater use in the edentulous group (87.3%) compared to dentate individuals (32.3%). Among those with upper dentures, 82.6% reported wearing them when eating, including a higher proportion of edentulous participants (87.4%) than dentate (77.4%). Removable lower dentures were reported by 37.2% overall, with use in 81.0% of edentulous and 19.2% of dentate participants (Table 2).

**Table 2:** Dental characteristics of dentate and edentulous participants (*N* = 566).

			**Dentate status**		
**Characteristic**, ***n (%) unless noted***		**All**	**Dentate 400 (71.7)**	**Edentulous 158 (28.3)**	***p*** **-value** ^†^
Removable upper denture (*n* = 559)	No	288 (51.5)	266 (67.7)	20 (12.7)	**< 0.0001**
	Yes	271 (48.5)	127 (32.3)	138 (87.3)	
Do you wear it when you eat? (*N* = 265)^a^	No	46 (17.4)	28 (22.6)	17 (12.6)	**0.0336**
	Yes	219 (82.6)	96 (77.4)	118 (87.4)	
Removable lower denture (*n* = 556)	No	349 (62.8)	315 (80.8)	30 (19.0)	**< 0.0001**
	Yes	207 (37.2)	75 (19.2)	128 (81.0)	
Do you wear it when you eat? (*N* = 202)^a^	No	41 (20.3)	17 (23.3)	23 (18.4)	0.4118
	Yes	161 (79.7)	56 (76.7)	102 (81.6)	
Treatment urgency (*n* = 390)	Urgent care	39 (7.2)	35 (9.0)	4 (2.7)	**< 0.0001**
	Early care	253 (46.7)	218 (55.9)	32 (21.6)	
	No obvious problem	250 (46.1)	137 (35.1)	112 (75.7)	
**Clinical Status of Dentate Participants**					
No. Upper teeth (*n* = 558)	*Mean (SD)*	5.8 (5.7)	8.0 (5.2)	–	–
	*Median (min, max)*	5.5 (0, 16)	9.0 (0, 16)	–	–
	Number with 0 upper teeth	236 (42.3)	78 (19.5)	–	–
No. Lower teeth (*n* = 557)	*Mean (SD)*	6.9 (5.3)	9.7 (3.5)	–	–
	*Median (min, max)*	8.0 (0, 16)	10.0 (0, 16)	–	–
	Number with 0 lower teeth	162 (29.1)	4 (1.0)	–	–
No. Total teeth (*n* = 558)	*Mean (SD)*	12.7 (10.4)	17.7 (7.9)	–	–
	*Median (min, max)*	13.0 (0, 32.0)	19.0 (1.0, 32.0)	–	–
	Number with 0 total teeth	158 (28.4)	0 (0)	–	–
Untreated decay (*n* = 394)	No	–	171 (43.4)	–	–
	Yes	–	223 (56.6)	–	–
Root fragment (*n* = 395)	No	-	264 (66.8)	–	–
	Yes	–	131 (33.2)	–	–
Periodontal conditions (*n* = 396)	No	-	231 (58.3)	–	–
	Yes	–	165 (41.7)	–	–

^a^For those who answered “yes” to having the specified type of denture.

^†^Likelihood ratio chi-square test; bold p-values indicate statistical significance at α= 0.05.

Participants self-reported how, when, and the justification for seeking or not seeking routine care (Table 3). Regarding dental service utilization, 1.8% reported never visiting a dentist, while 30.7% had seen a dentist within the past year. A five-year or more lapse since the last dental visit was reported by 32.9% overall, with a higher proportion in edentulous participants (47.7%) than dentate (27.1%) participants. Routine dental care in the last three years was reported by 34.9% overall, with 84.3% of edentulous and 55.1% of dentate participants not receiving such care.

**Table 3:** Dental service utilization and barriers to care (*N* = 566).

			**Dentate status**		
**Characteristic**, ***n(%)*** **unless noted**		**All**	**Dentate 400 (71.7)**	**Edentulous 158 (28.3)**	***p*** **-value** ^†^
Primary reason not visited dentist in past year (*N* = 392)	Cost	101 (25.8)	79 (28.4)	21 (19.4)	**0.0001**
	Limited or no access^a^	31 (7.9)	22 (7.9)	8 (7.4)	
	Do not know or have a regular dentist	12 (3.1)	8 (2.9)	4 (3.7)	
	Fear or apprehension^b^	21 (5.4)	16 (5.8)	5 (4.6)	
	No reason to go (no problems, no teeth)	102 (26.0)	52 (18.7)	47 (43.5)	
	Other reasons or priorities	102 (26.0)	42 (15.1)	13 (12.0)	
	2 or more factors^c^	69 (17.6)	59 (21.2)	10 (9.3)	
Main reason you last visited the dentist (*N* = 531)	Routine care, self-motivated	163 (30.7)	146 (38.8)	15 (10.2)	**< 0.0001**
	Treatment for previously identified problem	46 (8.7)	24 (6.4)	22 (15.0)	
	Called by dentist for routine care	22 (4.1)	16 (4.3)	6 (4.1)	
	Pain or irritation	159 (29.9)	122 (32.5)	34 (23.1)	
	Other reasons	126 (23.7)	53 (14.1)	70 (47.6)	
	2 or more factors^c^	15 (2.8)	15 (4.0)	0 (0)	
Time since last visit for any reason (*N* = 554)	Never	10 (1.8)	6 (1.5)	4 (2.6)	**< 0.0001**
	Past Year (< 12 mos. ago)	170 (30.7)	133 (34.0)	33 (21.3)	
	Between 1 & 2 years ago	95 (17.2)	77 (19.7)	17 (11.0)	
	Between 2 & 5 years ago	97 (17.5)	69 (17.7)	27 (17.4)	
	5 or more years	182 (32.9)	106 (27.1)	74 (47.7)	
During the past 3 years have you been to the dentist for routine cleanings (*N* = 551)	No	348 (63.2)	215 (55.1)	129 (84.3)	**< 0.0001**
	Yes	192 (34.9)	165 (42.3)	23 (15.0)	
	Don't know	11 (2.0)	10 (2.6)	1 (0.7)	
During the past 3 years how often have you been to the dentist for routine cleanings (*N* = 186^d^)	2+ times	71 (38.2)	66 (41.0)	3 (13.6)	**0.0289**
	1 time	48 (25.8)	38 (23.6)	9 (40.9)	
	Less than 1 time per year	25 (13.4)	23 (14.3)	2 (9.1)	
	When needed, not scheduled	42 (22.6)	34 (21.1)	8 (36.4)	
Gone to the ER or hospital for dental pain (*N* = 547)	No	517 (94.5)	365 (94.3)	146 (95.4)	0.6007
	Yes	30 (5.5)	22 (5.7)	7 (4.6)	
Is there a particular dentist that you usually go to for clinical care (*N* = 546)	No	241 (44.1)	187 (48.8)	79 (51.0)	0.1468
	Yes	269 (49.3)	175 (45.7)	61 (39.4)	
	Don't know	36 (6.6)	21 (5.5)	15 (9.7)	

^a^Cannot access the office or clinic due to distance, no transportation, no appointment availability.

^b^Fear, apprehension, nervousness, pain, dislike going.

^c^2 or more of the listed factors above.

^†^Likelihood ratio chi-square test; bold p-values indicate statistical significance at α= 0.055.

^d^Of those who answered “Yes,” they visited the dentist for routine cleanings in the past 3 years.

Most participants did not visit a dentist in the previous year due to prohibitive cost, not having a need to do so, or some combination of reasons; this did differ by dentate status, and dentate participants were more likely to not seek care due to cost, while more edentulous participants did not seek care due to lacking a reason. Recent dental visits were largely for routine care, pain/irritation, or some other reasons; more dentate participants visited the dentist for routine care or pain/irritation, compared to edentulous. More edentulous participants last visited a dentist for treatment of previously identified problems or other reasons. Most participants last visited the dentist either in the previous 12 months, or more than 5 years previously; dentate participants tended to have more recent visits than edentulous (previous 12 months, between 1–2 years, between 2–5 years). More edentulous participants had not visited a dentist in 5 or more years compared to dentate. More dentate participants had visited the dentist for routine cleaning in the previous 3 years, compared to edentulous. Of those who had visited the dentist in the previous 3 years, most visited at least one time per year. Dentate participants did not consistently visit the dentist more or less than edentulous participants; in general, dentate participants reported visiting the dentist more twice per year compared to edentulous participants, while more edentulous participants reported visiting once per year as-needed. Participants did not differ by dentate status according to whether or not they sought care at an Emergency Room (ER)/hospital for dental pain, or if they have a regular dental provider (Table 3).

There was a difference in the time since last seeking dental care; edentulous participants were more likely to have an increased time since their last visit. Specifically, more edentulous participants had 5 or more years since their last visit, and dentate participants were more likely to have had a more recent visit (less than 5 years). More dentate participants had sought routine care in the previous 3 years compared to edentulous participants. More dentate participants required Early or Urgent Care compared to edentulous participants; conversely, more edentulous participants had No Obvious Problem, compared to dentate participants (Table 3).

**Research question 2** *: What are the barriers for access to oral care of older adults in central Alabama, as indicated by not receiving routine care in the previous 3 years?*

Edentulous status and region of residence (metro, non-metro) were associated with the likelihood of not receiving routine care in the previous 3 years. Dentate participants were more likely to receive routine care, compared to edentulous participants (*AOR* = 4.04, 95% CI: 2.33, 6.99). Those living in a metro region were less likely to receive routine care compared to those living in a non-metro region (*AOR* = 0.60, 95% CI: 0.38, 0.95; Table 4).

**Table 4:** Model results for logistic regression.

**Model outcome (*N*^a^)**	**Predictor**	**Level**	***AOR* ^b^**	**95%*CI*^c^**	***p*-value^d^**
Received routine care in previous 3 years or check-up (yes vs. no) (*N* = 396)	Dentate status	Edentulous vs. dentate	4.04	2.34, 6.99	< 0.0001
	Region of residence	Metro vs. non-metro	0.60	0.38, 0.95	0.0288
Untreated decay (yes vs. no) (*N* = 284)	Race	Black/African American vs. White	1.89	1.15, 3.11	0.0125
	Driving status	No vs. yes	1.97	1.12, 3.48	0.0196
	Dental insurance	No vs. yes	1.85	1.13, 3.02	0.0143
Number of natural teeth remaining (0, 1–19, 20–32) (*N* = 403)	Received routine care in previous 3 years or check-up	No vs. yes	3.66	2.44, 5.49	< 0.0001
		Unsure vs. yes	1.01	0.24, 4.32	
	Age	-	1.04	1.02, 2.07	0.0011

^a^Number of participants included in the model due to having complete predictor and outcome measures.

^b^Adjusted odds ratio.

^c^Wald 95% confidence interval.

^d^Type 3 effect test, Wald.

**Research question 3**: *What are the factors related to untreated decay, as an indicator of unmet oral health needs?*

Of dentate participants, race, driving status, and dental insurance status were associated with untreated decay. Black or African American participants were more likely than White participants to have untreated decay (*AOR* = 1.89, 95% CI: 1.15, 3.11). Those who do not drive were more likely to have untreated decay compared to those who do (*AOR* = 1.97, 95% CI: 1.12, 3.48). Those without dental insurance were more likely to have untreated decay compared to those who do (*AOR* = 1.85, 95% CI: 1.31, 3.02; Table 4).

**Research question 4**: *What are the factors related to number of natural teeth retained?*

Among all participants, 158 (28.4%) had 0 teeth remaining; 215 (38.5%) had 1–19 teeth remaining (moderate to severe tooth loss); and 185 (33.2%) had 20–32 teeth remaining (functional dentition). Age and receiving routine care in the last 3 years are associated with the number of natural teeth remaining (LR Chi-square (DF = 3) = 50.23, *p* < 0.0001). The positive estimate for age indicates an increased probability of having fewer teeth as age increases (*AOR* = 1.04, 95%CI: 1.02, 2.07). Those who had not received routine care in the previous 3 years were more likely to have fewer natural teeth compared to those who had received routine care (*AOR* = 3.66, 95%CI: 2.44, 5.49; Table 4). There was not a difference between those who were unsure if they had received routine care.

**Research question 5**: *What are the factors related to treatment urgency?*

Race, screening site, residential region, dental insurance status, and dentate status were associated with treatment urgency (Early or Urgent Care or No Obvious Problem) (LR Chi-square (*DF* = 6) = 120.29, *p* < 0.0001). There were 391 participants included in the model due to having complete predictor and outcome measures (Table 5).

**Table 5:** Model results for binomial logistic regression, predicting treatment urgency (*N* = 391).

**Predictor**	**Comparison**	**Estimate (*SE*)^a^**	***AOR* ^b^**	**95%*CI*^c^**	***p*-value^d^**
Race	Female vs. male	0.89 (0.28)	–	–	0.0015
Site	Living facility vs. senior center	0.94 (0.31)	2.54	1.40, 4.63	0.0023
Region	Metro vs. non–metro	0.85 (0.30)	2.33	1.30, 4.20	0.0047
Dentate status	Edentulous vs. dentate	−1.24 (0.44)	–	–	0.0054
Dental Insurance	No vs. yes	0.63 (0.24)	1.88	1.16, 3.03	0.0101
Race*Dentate status		−1.52 (0.56)	–	–	0.0068
	Edentulous vs. dentate, Black/African American	–	0.06	0.03, 0.13	–
	Edentulous vs. dentate, White	–	0.29	0.12, 0.69	–

^a^Maximum likelihood estimates, standard error.

^b^Adjusted Odds Ratio.

^c^Wald 95% Confidence Interval.

^d^Type 3 Effect test, Wald.

Those screened in a living facility were more likely than those screened in a senior center to require some care (Early or Urgent care) compared to having No Obvious Problem (*AOR* = 2.54, 95%CI: 1.40, 4.63). Those in a metro area were more likely to require some care compared to those in a non-metro area (*AOR* = 2.33, 95%CI: 1.30, 4.20).

**Figure 1:**
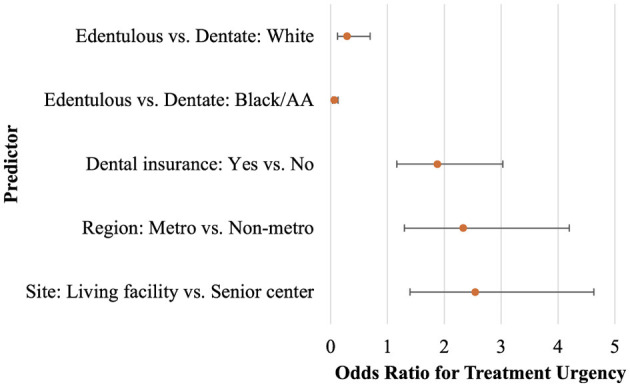
Odds ratios for treatment urgency (Early or Urgent care vs. No obvious problem), with 95% Wald Confidence Intervals; produced from binomial regression predicting treatment urgency.

Those with no dental insurance were more likely to require some care (*AOR* = 1.88, 95%CI: 1.16, 3.03) compared to those with dental insurance. The interaction between race and dentate status was statistically significant. While all patients, regardless of race, were less likely to be edentulous and more likely to be dentate, the degree varied with race. Those who were Black or African American were 94.0% less likely to be edentulous compared to dentate (*AOR* = 0.06, 95%*CI* = 0.03, 0.13); those who were White were 71% less likely to be edentulous compared to dentate (*AOR* = 0.29, 95%*CI* = 0.12, 0.69; Table 5, Figure 1).

## Discussion

Longevity is a topic commonly discussed but quality of life must be examined as populations live longer. The southern US region is home to 38% of the US population that is 65 and older (30). There is a need to better understand the oral health needs of older adults both in urban and rural areas, particularly those who experience low income and lack access to dental care. This program is designed to promote oral health awareness, determine the oral health status, and assess needs and barriers to care among seniors. This community-based collaboration creates a sustainable network of stakeholders who support engagement with older adults (from State policy and programming, to academic experiences, to living spaces and senior centers, to residents and their families in the communities served). Unique characteristics of the partnership include participant education on their oral health status, engagement with (relatively young) dental trainees and connecting older adults living in Alabama to oral health care resources in their area. This oral health needs inventory is essential as a needs assessment and to entice recommendations for services in support of those who are aging in place in the state. The United Health Foundation (US Census Bureau dataset of 2023) reports that 46.9% of adults 65 or older residing in Alabama live in rural areas (31).

Our study identifies several key findings regarding the oral health status and barriers to care among older adults in central Alabama. We observed high rates of untreated caries and edentulism. The association between treatment urgency and metropolitan residence, as well as the impact of dental insurance and time since last visit on tooth loss, are notable outcomes. Additionally, the most common reason for not seeking regular dental care among edentulous participants was a perceived lack of need, and driving status emerged as a potential indicator of access to oral health services. These findings will be discussed in detail in the following paragraphs.

The prevalence of edentulism in our sample of older adults in central Alabama was 28.3%, which is higher than the median prevalence of 17% reported for community-dwelling older adults in the 2011–2016 National Health and Nutrition Examination Survey (NHANES) across 10 states (32). Recent national data indicate that rates of complete tooth loss among older adults living below the federal poverty level range from 22.9% to 30%, with higher rates observed among Black or African American and rural populations (33, 34). Our findings are consistent with these estimates for disadvantaged groups, and also align with secular trends showing persistent socio-economic disparities in edentulism in the United States (35, 36). These results suggest that older adults in central Alabama experience edentulism at rates similar to the most vulnerable populations in the United States.

Untreated dental caries among dentate older adults in our study were highly prevalent, with a rate of 39% compared to the median prevalence of 16% in the 2011–2016 NHANES data across 11 states (32). National estimates report that 29–35% of non-Hispanic Black and low-income older adults have untreated caries, compared to 16–21% in the general population (37–39). Our findings are consistent with national trends showing that low-income and underrepresented populations bear a disproportionate burden of untreated decay, as confirmed by Lin et al., 2019 and Bashir, 2022 using NHANES and other large-scale surveys (32, 39). The prevalence of untreated caries and the barriers to care, such as financial concerns, are similar to those reported in previous studies from other countries, further highlighting the global challenge of oral health disparities among older adults (37–40).

Participants living in metro areas were less likely to receive routine care than those living in non-metro region, a finding that contrasts with other studies reporting more difficulty with transportation in rural areas and, by extension, less access to health care (41, 42). Birmingham is the largest city in Alabama; however it is lacking a robust public transportation system. Other studies have shown associations between lower life expectancy and other health indicators within metro areas across Alabama (43, 44). Additional research is needed to examine interactions with oral health indicators at the county level, as well as the nature of access to other modes of transportation other than personal vehicles.

This initial survey data reveals that increased education was not consistent with increased likelihood of receiving routine preventative dental care which emphasizes the need for oral health education and connecting older adults to oral care providers to help them maintain their natural dentition, periodontal health and to contribute to overall wellness.

In this study, treatment urgency (early or urgent care) was associated with metro areas; an observational study in another southern state, Texas, found no difference in oral health status based on rural/urban areas (45). Lack of dental insurance, as well as time since last dental visit, were also associated with tooth loss in a study among older adults in rural Colorado (46). Further research, with a larger sample size, is needed to explore potential differences between rural and urban areas in Alabama.

The most common reason for not visiting a dentist regularly among edentulous participants was lack of a reason or problem; a concerning finding which highlights the need to raise awareness for regular examination by a dental professional for early detection and prevention of more invasive (and more costly) treatments later. Evaluation of driving status as an indicator for oral health status or utilization of preventive services is lacking in the literature; further research is needed in this area as it relates to access to oral health services. Mobility, transportation and oral health workforce availability are significant barriers to ponder when caring for older adults.

We identified limitations related to data collection and results. While this work attempts to fill a gap in knowledge for Alabama, our results are geographically limited to central Alabama, within an hour's diving distance from the University of Alabama at Birmingham (UAB), restricting generalizability. Due to lack of identifying details recorded, we cannot confirm that we do not have multiple responses from the same person; we expect this occurrence to be low and not unduly influence statistical results. The screener was not the same person before and after 2020 (treatment urgency and active disease); this inconsistency might influence results if we see a difference related to specifying treatment urgency and active disease. Finally, root fragments were counted as a tooth.

Some issues were encountered due to incomplete data contributing to small sample size for certain analyses. Because it was of interest to learn about the degree of dental care required (none, early, urgent), the three levels were initially modeled. However, due to the relatively smaller number of participants requiring urgent care in the model (*N* = 27 *who had data for predictors included in the model*), relative to early care (*N* = 179) or no care (*N* = 185), wider confidence intervals were observed for screening site. Therefore, treatment urgency was ultimately modeled by dichotomizing treatment urgency. Larger sample sizes could again provide insight into the degree of dental care required. Wide confidence intervals were also seen when modeling the number of remaining teeth relative to complete data for predictors, although not to the same magnitude, so the modeled number of ordinal groups of teeth were appropriate.

Through this analysis, the resulting community-based needs assessment can be used to quantify oral health needs and correlate the data identifying barriers to care (including social determinants of health) for the older adult population in central Alabama. Dissemination of this information raises awareness of oral health needs among stakeholders and partners throughout the State, inviting policy makers to plan and direct resources to strengthen future programs and assistance through evidence-based practice allowing for resources to be utilized where they are needed the most and where they have the greatest impact on improving health and wellbeing. This work (1) improves surveillance and enhances the knowledge base to inform and guide the planning and development of enhanced educational programming specific to older adults, caregivers and staff members at living facilities, and allied health professionals, (2) raises awareness of preventive and treatment needs related to oral and overall health, and (3) enhances capacity for connecting seniors to resources in their area. This model serves as a template for other areas to potentially replicate successes of this academic-community partnership—and establish systems, processes, and tools—to expand projects to other sites (e.g., older adult living communities, senior centers). An extension of this work is planned to investigate geographic distance from point of care. Future studies could focus on longitudinal surveillance and analysis to guide public health community-based initiatives to continuously serve the residents based on their specific needs. Potential extension of this study includes investigating the relationship between total number of teeth and frailty.

## Data Availability

The raw data supporting the conclusions of this article will be made available by the authors, without undue reservation.
